# Relationships of handgrip strength with the presence of cerebral microbleeds and platelet count in older Japanese adults

**DOI:** 10.18632/oncotarget.27573

**Published:** 2020-05-12

**Authors:** Hirotomo Yamanashi, Kenji Nagaoki, Sinsuke Kanbara, Yuji Shimizu, Kunihiko Murase, Akira Tsujino, Takahiro Maeda

**Affiliations:** ^1^Department of General Medicine, Nagasaki University Graduate School of Biomedical Sciences, Sakamoto, Nagasaki, Japan; ^2^Department of Infectious Diseases, Nagasaki University Hospital, Sakamoto, Nagasaki, Japan; ^3^Department of Clinical Medicine, Institute of Tropical Medicine, Nagasaki University, Sakamoto, Nagasaki, Japan; ^4^Nagasaki Prefecture Goto Central Hospital, Goto, Nagasaki, Japan; ^5^Department of Community Medicine, Nagasaki University Graduate School of Biomedical Sciences, Sakamoto, Nagasaki, Japan; ^6^Department of Neurology and Strokology, Nagasaki University Hospital, Sakamoto, Nagasaki, Japan; ^7^Department of Island and Community Medicine, Nagasaki University Graduate School of Biomedical Sciences, Goto, Nagasaki, Japan

**Keywords:** sarcopenia, frailty, handgrip strength, cerebral microbleeds, platelet

## Abstract

Introduction: Lower handgrip strength is a manifestation of sarcopenia and frailty, and has been reported to be associated with cerebral microbleeds (CMBs), which appear on T2^*^-weighted magnetic resonance scans as low-intensity spots. However, the underlying mechanism is unknown. We hypothesized that vascular endothelial injury could be the common factor in loss of handgrip strength and CMBs. We aimed to clarify the relationship between handgrip strength and CMBs, with reference to a marker of vascular repair capability.

Materials and Methods: We conducted a cross-sectional study of 95 60- to 87-year-old Japanese people who underwent brain magnetic resonance imaging in 2016–2017. Baseline information was obtained by trained interviewers regarding the age, sex, smoking status, nutrient intake, cognition, medical history, education, and household income of the participants. Physical activity was assessed using a tri-axial accelerometer. We used the Fried frailty phenotype definition. Multivariable linear regression analysis was performed.

Results: Handgrip strength was independently associated with the presence of CMB after adjustment for age, sex, body mass index, classical cardiovascular risk factors, protein intake, and daily activity (B = −3.43, *p* = 0.027). This association was shown in participants with a low (B = −4.05, *p* = 0.045) but not high platelet count (B=−2.23, *p* = 0.479). Frailty was also independently associated with the presence of CMB after adjustment for confounders (B = 0.57, *p* = 0.014). Although this association was not present in participants a high platelet count, there was a positive trend in those with a low platelet count (B = 0.50, *p* = 0.135).

Conclusions: Platelet count, a marker of vascular repair capability, appears to modify the relationship between handgrip strength and CMBs.

## INTRODUCTION

Low handgrip strength is a typical manifestation of sarcopenia and frailty [1, 2], and is itself a predictor of mortality and disability [3, 4]. Our previous studies have shown that the capacity for endothelial repair is associated with the maintenance of handgrip strength [5, 6]. Both platelets and CD34-positive cells play important roles in vascular homeostasis [7–9], respond to hypertension-induced vascular endothelial injury, and maintain handgrip strength. Platelets may also increase the proliferation of bone marrow multi-potent stem cells [10] and induce the differentiation of human CD34-positive cells into foam cells [11], which leads to the development of atherosclerotic lesions as a consequence of aggressive repair in response to vascular damage.

Recent studies have shown that small vessel disease is associated with frailty [12–15]. Cerebral microbleeds (CMBs) are a manifestation of small vessel disease and are visible on T2^*^-weighted magnetic resonance imaging (MRI) scans as low-intensity spots [16]. Hypertension is the most consistent predictor of CMBs, with an odds ratio in healthy adults of 4.21 (95% confidence interval 2.20–8.08) [17]. In a cohort of 962 Asian adults, the presence of CMBs was significantly associated with physical frailty and low handgrip strength, independent of age, sex, and vascular risk factors [14]. In addition, of the five assessments of frailty used, handgrip strength was the only one to be associated with brainstem CMBs after multivariate adjustment. However, CMBs were not significantly associated with frailty after adjustment for age, sex, and duration of education in a cohort of 388 Australian adults [13].

Platelets are initiators of vascular inflammation and vessel wall remodeling [18, 19], and age-related increases in inflammatory agents can disrupt the microvascular endothelium. In turn, disruption of the microvascular endothelium impairs the microcirculation and prevents delivery of the oxygen and nutrients necessary to maintain skeletal muscle mass [20]. Moreover, failure of the cerebrovascular endothelium is the underlying mechanism of cerebral small vessel disease, including CMBs [21]. Because the platelet count has a crucial role in vascular endothelial repair activity [5] and may be a common etiologic factor in the loss of handgrip strength and CMBs, we hypothesized that the relationships between handgrip strength, frailty, and CMBs might be more marked in older people with low platelet counts. To clarify these relationships, we conducted a cross-sectional study of 95 Japanese people aged 60–87 years who underwent brain MRI in 2016–2017.

## RESULTS

Table 1 lists the characteristics of the whole cohort and groups categorized according to their platelet count. Of the 95 participants, 18 (19.0%) had CMBs in any location, 12 (12.6%) had CMBs in lobar and/or other locations, and 13 (13.7%) had CMBs in non-lobar and/or lobar locations. According to Fried Frailty phenotyping, there were 40, 50, and 5 non-frail, pre-frail, and frail participants, respectively. Compared with those participants with a high platelet count, those with a low platelet count were more likely to have chronic kidney disease (CKD) and use non-vitamin K antagonist oral anticoagulants (NOACs) more frequently, had a higher serum cystatin C concentration, and had a lower low-density lipoprotein (LDL)-cholesterol concentration.

**Table 1:** Clinical characteristics of the study cohort, categorized according to platelet count

	All	Low platelet count	High platelet count	*P*-value
No. of participants	95	48	47	
Age, years	72.4 ± 7.3	73.8 ± 7.3	71.0 ± 7.0	0.061
Men	48 (50.5)	25 (52.1)	23 (48.9)	0.759
Height, cm	155.5 ± 8.7	155.7 ± 8.7	155.4 ± 8.7	0.873
Body mass, kg	61.6 ± 13.2	62.0 ± 14.4	61.3 ± 12.0	0.870
Body mass index, kg/m^2^	25.4 ± 4.6	25.4 ± 4.5	25.4 ± 4.7	0.953
Handgrip strength, kg	26.6 ± 9.3	25.5 ± 9.7	27.7 ± 8.8	0.245
Systolic blood pressure, mmHg	136.1 ± 20.9	136.3 ± 21.3	135.9 ± 20.6	0.685
Diastolic blood pressure, mmHg	77.4 ± 12.5	77.1 ± 14.1	77.6 ± 10.7	0.985
History of ischemic heart disease	10 (10.5)	6 (12.5)	4 (8.5)	0.526
Hypertension	64 (67.4)	30 (62.5)	34 (72.3)	0.306
Diabetes mellitus	27 (28.4)	14 (29.2)	13 (27.7)	0.871
Dyslipidemia	44 (46.3)	19 (39.6)	25 (53.2)	0.184
Atrial fibrillation	10 (10.5)	6 (12.5)	4 (8.5)	0.526
Chronic kidney disease	9 (9.5)	8 (16.7)	1 (2.1)	**0.016**
Use of antiplatelet and/or anticoagulant medication	26 (27.4)	17 (35.4)	9 (19.2)	0.075
NOACs^†^	13 (13.7)	10 (20.8)	3 (6.4)	**0.040**
Warfarin	3 (3.2)	1 (2.1)	2 (4.3)	0.545
Use of antiplatelet medication	14 (14.7)	8 (16.7)	6 (12.8)	0.592
White blood cell count, cells/μL	5,309 ± 1609	5,319 ± 1544	5,704 ± 1666	0.255
Platelet count, ×10^4^/μL	20.7 ± 5.0	16.7 ± 3.0	24.7 ± 2.9	**< 0.001**
Total protein, g/dL	7.1 ± 0.5	7.0 ± 0.5	7.2 ± 0.5	0.066
Albumin, g/dL	4.3 ± 0.4	4.2 ± 0.4	4.3 ± 0.3	0.260
High-sensitivity C-reactive protein, g/dL	1,303 ± 3123	1,328 ± 3670	1,277 ± 2484	0.443
Cystatin C, mg/dL	1.05 ± 0.27	1.11 ± 0.28	1.00 ± 0.24	**0.038**
Hemoglobin A1c, %	5.94 ± 0.77	5.89 ± 0.77	5.98 ± 0.78	0.657
Total cholesterol, mg/dL	183 ± 35	178 ± 42	188 ± 26	0.120
HDL-cholesterol, mg/dL	56 ± 17	58 ± 20	54 ± 14	0.522
LDL-cholesterol, mg/dL	100 ± 26	94 ± 29	106 ± 22	**0.017**
Triglycerides, mg/dL	125 ± 66	122 ± 77	128 ± 54	0.129
Smoking status				0.590
Never	46 (48.4)	21 (43.8)	25 (53.2)	
Former	41 (43.2)	22 (45.8)	19 (40.4)	
Current	8 (8.4)	5 (10.4)	3 (6.4)	
Duration of education, years				0.829
6–9	54 (56.8)	26 (54.2)	28 (59.6)	
10–12	29 (30.5)	16 (33.3)	13 (27.7)	
≥ 13	12 (12.6)	6 (12.5)	6 (12.8)	
Household income, million yen per year (US dollars per year)			0.370
< ¥200 ($17,544)	36 (37.9)	18 (37.5)	18 (38.3)	
¥200–399 ($17,544–35,088)	51 (53.7)	28 (58.3)	23 (48.9)	
¥400–599 ($35,088–52,632)	6 (6.3)	2 (4.2)	4 (8.5)	
¥600–799 ($52,632–70,175)	0 (0)	0 (0)	0 (0)	
≥ ¥800 ($70,175)	2 (2.1)	0 (0)	2 (4.3)	
Mini-Mental State Examination score	25.8 ± 2.8	25.5 ± 2.4	26.2 ± 3.1	0.062
Center for Epidemiologic Studies Depression Scale score	7.1 ± 6.4	8.5 ± 7.4	5.6 ± 4.8	0.068
Daily energy expenditure during physical activity, kcal	500 ± 159	499 ± 156	503 ± 172	0.378
Daily energy intake, kcal	1824 ± 564	1855 ± 429	1792 ± 677	0.217
Daily dietary protein intake, g	76.4 ± 27.5	77.5 ± 22.2	75.4 ± 32.3	0.330
Daily dietary vitamin D intake, μg	20.2 ± 14.6	20.3 ± 13.7	20.0 ± 15.7	0.639
Daily dietary alcohol intake, g	6.0 ± 16.9	5.5 ± 14.6	6.5 ± 19.0	0.647
Presence of cerebral microbleeds	18 (19.0)	12 (25.0)	6 (12.8)	0.128
Number of cerebral microbleeds	0.5 ± 1.4	0.6 ± 1.5	0.4 ± 1.2	0.130
Fried Frailty phenotype				0.278
Non-frail	40 (42.1)	20 (41.7)	20 (42.6)	
Pre-frail	50 (52.6)	24 (50.0)	26 (55.3)	
Frail	5 (5.3)	4 (8.3)	1 (2.1)	
Low handgrip strength	24 (25.3)	15 (31.3)	9 (19.2)	0.175
Low activity	9 (9.5)	5 (10.4)	4 (8.5)	0.524
Slow walking speed	17 (17.9)	10 (20.8)	11 (23.4)	0.763
Exhaustion	14 (14.7)	7 (14.6)	7 (14.9)	0.966
Weight loss	9 (9.5)	4 (8.3)	5 (10.6)	0.701

Data are mean ± standard deviation or *n* (%). ¥114 was equivalent to $1 on March 4, 2016. ^†^NOACs: non-vitamin K antagonist oral anticoagulants (dabigatran, rivaroxaban, apixaban, or edoxaban). LDL: low-density lipoprotein; HDL: high-density lipoprotein.

In the simple linear regression analysis, although the association between handgrip strength and the presence of CMBs was not significant, there was a negative trend among the total participants (B coefficient = −3.00, *p* = 0.218) (Table 2). Frailty was also independently associated with the presence of CMBs among the total participants (B = 0.65, *p* = 0.004).

**Table 2:** Multivariable linear regression analysis of the relationship between handgrip strength and the presence of cerebral microbleeds

	All (*n* = 95)			Low platelet count (*n* = 48)			High platelet count (*n* = 47)		
	B coefficient	95% confidence interval	*P*-value	B coefficient	95% confidence interval	*P*-value	B coefficient	95% confidence interval	*P*-value
Handgrip strength									
Crude	−3.00	(−7.80, 1.81)	0.218	−2.57	(−9.09, 3.95)	0.431	−2.67	(−10.44, 5.10)	0.493
Model 1	−3.28	(−6.24, −0.32)	**0.030**	−3.80	(−7.74, 0.14)	0.059	−1.75	(−6.96, 3.46)	0.501
Model 2	−3.57	(−6.59, −0.55)	**0.021**	−3.97	(−7.70, −0.25)	**0.037**	−1.57	(−7.88, 4.73)	0.616
Model 3	−3.43	(−6.45, −0.41)	**0.027**	−4.05	(−8.02, −0.09)	**0.045**	−2.23	(−8.58, 4.11)	0.479
Frailty									
Crude	0.65	(0.21, 1.08)	**0.004**	0.64	(0.01, 1.27)	**0.046**	0.65	(−0.02, 1.32)	0.057
Model 1	0.64	(0.21, 1.06)	**0.004**	0.62	(0.02, 1.21)	**0.043**	0.70	(0.00, 1.40)	**0.049**
Model 2	0.60	(0.15, 1.05)	**0.009**	0.59	(−0.04, 1.22)	0.065	0.69	(−0.14, 1.51)	0.101
Model 3	0.57	(0.12, 1.02)	**0.014**	0.50	(−0.16, 1.17)	0.135	0.45	(−0.35, 1.26)	0.262

Model 1 was adjusted for age, sex, and BMI. Model 2 was adjusted for the Model 1 factors and classical cardiovascular risk factors (history of hypertension, history of diabetes mellitus, cystatin C, and smoking status). Model 3 was adjusted for the Model 2 factors and risk factors for sarcopenia (protein intake, vitamin D intake, and daily activity).

In the multivariable linear regression analysis, handgrip strength was found to be independently associated with the presence of CMBs in all the models tested (B = −3.44, *p* = 0.027 in Model 3) (Table 2). This association was shown in the low platelet count group (B = −4.05, *p* = 0.045 in Model 3), but not in the high platelet count group (B = −2.23, *p* = 0.479 in Model 3). Frailty was also independently associated with the presence of CMBs in all the models tested (B = 0.57, *p* = 0.014 in Model 3). Although this association was not shown in the participants when they were categorized according to platelet count, there was a positive trend in the low platelet count group (B = 0.50, *p* = 0.135 in Model 3).

We additionally performed multivariable linear regression analyses of the relationship between handgrip strength and the presence of CMBs by sex and by age group. Although this association was not statistically significant in men, a negative trend was found in both men and women in Model 3 (B = −1.66, *p* = 0.529 and B = −5.11, *p* = 0.005, respectively). The age categories were defined as young (60–71 years) and old (72–87 years) using a median age of 71 years. Although these associations were not statistically significant, a negative trend was found in both the young and old age groups in Model 3 (B = −4.35, *p* = 0.054 and B = −1.36, *p* = 0.532, respectively).

The number of CMBs was not associated with either handgrip strength or frailty in all patients, the low platelet count group, or the high platelet count group in Model 3. When we selected the distribution of CMBs as only lobar CMBs, the association between handgrip strength and CMBs was attenuated to a non-significant level but remained significant in non-lobar CMBs (B = −1.02, *p* = 0.604 and B = −3.57, *p* = 0.037 in Model 3, respectively). The association between frailty and lobar CMBs and the association between frailty and non-lobar CMBs were attenuated to a non-significant level (B = 0.25, *p* = 0.404 and B = 0.31, *p* = 0.233 in Model 3, respectively).

## DISCUSSION

The main findings of the present study were that handgrip strength is significantly associated with the presence of CMBs, but that this association is only present in people with a relatively low platelet count. Frailty was also shown to be associated with the presence of CMBs, although this association was not significant in people with low platelet counts. These results demonstrate that frailty and its manifestation, low handgrip strength, and CMBs may have vascular dysfunction as a common etiologic factor because the association between the two was strong in participants with low platelet counts.

This is the first epidemiologic study to show that the association between handgrip strength and the presence of CMBs can be stratified according to platelet count in an older Japanese population without a history of stroke or dementia. The findings were strengthened by adjustment for the potential confounders of cardiovascular risk factors, nutritional status, socio-economic status, and physical activity.

Previous studies have shown that small vessel disease is associated with frailty [12–15]. A cohort study of 791 older adults in the US showed the progression of frailty in > 95% during up to 14 years of follow-up [15]. In mixed-effect analysis, arteriosclerosis, identified during postmortem brain examination, was associated with the progression of frailty after controlling for the effects of age, sex, education, and their interaction with time (estimate = 0.079, *p* < 0.001). The authors suggested that frailty and brain pathology might be caused by a common underlying pathophysiologic mechanism. To the best of our knowledge, few epidemiologic studies have examined the underlying pathophysiology. However, a cross-sectional study of Taiwanese adults showed that the number of CMBs is associated with the degree of physical frailty and that CMBs located in the brainstem are significantly associated with physical frailty and low handgrip strength [14]. Because the location of CMBs reflects the underlying mechanism (deep and/or infratentorial CMBs are attributable to hypertensive arteriosclerosis, whereas lobar CMBs are attributable to cerebral amyloid angiopathy [21–24]), CMBs located in the brainstem may be indicative of disruption of the microcirculation. Thus, the findings of previous studies indicate that the associations between physical frailty or low handgrip strength and CMBs may be underpinned by common defects in the microcirculation. The findings in our study showed that CMBs in any location and non-lobar CMBs were significantly associated with handgrip strength, but lobar CMBs were not. These findings are compatible with the hypothesized underlying mechanism in non-lobar CMBs, which may be different from cerebral amyloid angiopathy in lobar CMBs.

**Figure 1:**
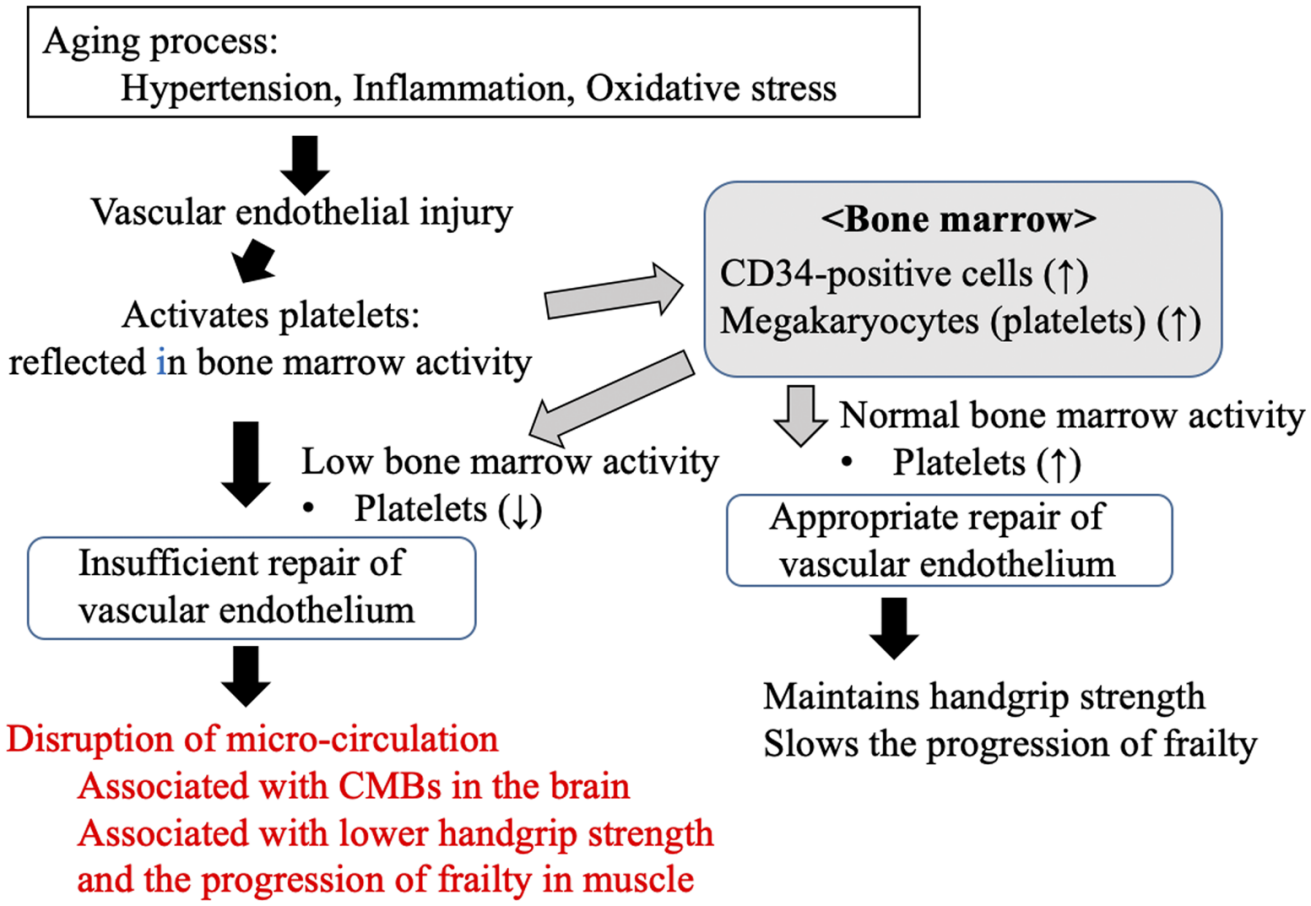
Possible mechanism underlying the links between the progression of frailty, handgrip strength, cerebral microbleeds, and platelet count, as an index of endothelial repair capability, in older people in the present study. CMBs: cerebral microbleeds.

Platelets play an important role in vascular inflammation and vessel wall remodeling [18, 19], and our previous study showed that the platelet count is a marker of vascular repair capability in older Japanese men [5]. Activated platelets can increase the proliferation of bone marrow mesenchymal stem cells [10] and the number of CD34-positive cells by releasing stromal cell-derived factor 1α [25]. These cells, in turn, can also contribute to endothelial repair [9]. The presence of sufficient numbers of platelets may enhance the repair of the vascular endothelium. However, low bone marrow activity may limit the proliferation of platelets, leading to poor endothelial repair. Disruption of the microvascular endothelium impairs the microcirculation [20]. Thus, people with a low platelet count may be at higher risk of microcirculatory dysfunction in the brain, leading to CMBs, and in muscle, leading to lower handgrip strength and the progression of physical frailty (Figure 1).

Inflammation is also involved in the induction of vascular endothelial injury; therefore, we adjusted Model 3 in the multivariate analysis according to the serum concentration of high-sensitivity C-reactive protein (hs-CRP). However, both the associations between handgrip strength and the presence of CMBs (B = −3.40, *p* = 0.031) and frailty and the presence of CMBs (B = 0.61, *p* = 0.010) remained, which suggests that the underlying pathophysiology principally involves disruption of the microcirculation and insufficient vascular endothelial repair, rather than inflammation.

### Study strengths and limitations

Our study had a strength. The muscle strength and CMBs had predisposing factors, and they should be considered potential confounding factors. We collected detailed information about cardiovascular risk factors, inflammation, nutritional status, physical activity level, socioeconomic status, and other parameters.

The potential limitations of the present study warrant some discussion. First, because we recruited participants after an MRI examination had been performed, the prevalence of CMBs may have been underestimated. Second, the purpose of brain MRI in most patients was to examine somatic symptoms, and eligible participants who needed chronic care were recruited at a hospital visit; therefore, the participants might have had a higher probability of being frail than the normal population. However, despite this potential selection bias, the risk of confounding of the association might have been low in this study. In addition, because this was a cross-sectional study, we were unable to establish cause–effect relationships.

In conclusion, the platelet count, a marker of vascular repair capability, appears to modify the association between handgrip strength and the presence of CMBs in older people. Further study is required to elucidate the reason why the association between CMBs and the maintenance of muscle strength depends on the platelet count, and may help to identify preventive strategies for frailty and sarcopenia.

## MATERIALS AND METHODS

### Ethics statement

This study was approved by the Ethics Committee of Nagasaki University Graduate School of Biomedical Sciences (project registration number 15122574) and by the Ethics Committee of Nagasaki Goto Central Hospital (project registration number 27-1), and was conducted according to the ethical standards defined in the 1964 Declaration of Helsinki and its subsequent amendments.

### Study design and participants

We conducted a cross-sectional study in Nagasaki Goto Central Hospital, Japan. We recruited all the patients aged 60 years or older who underwent plain brain MRI using T2^*^-weighted gradient-recalled echo sequence imaging. The purpose of examining brain MRI was as follows; dizziness, vertigo, syncope, headache, unconsciousness, seizure, loss of balance, numbness, suspected stroke or suspected dementia.

The participants were sequentially recruited from the MRI list between January 1, 2013 and December 31, 2015, when they attended a regular consultation at the hospital. We did not include patients who had been diagnosed with or treated for stroke or dementia. Of 1,612 patients who underwent MRI, 111 were included, and the rest of the patients were not recruited because of shortage of funding. Ultimately, 95 participants (48 men and 47 women) with a mean age of 72.4 years (standard deviation, 7.3 years; range, 60–87 years) were evaluated, after the exclusion of patients with a refusal to participate (*n* = 9), a metal artifact on MRI (*n* = 1), a history of stroke or dementia recognized at the interview conducted after the recruitment process (*n* = 3), and refusal to use an accelerometer (*n* = 3). All the participants provided their written informed consent at the time of their regular visit, between March 4, 2016 and March 2, 2017. All the anthropometric data, medical records, and laboratory data, with the exception of the MRI data, were obtained after the provision of informed consent.

### Definition of CMBs

Multi-sequence MRI examinations were performed using a 1.5-T scanner (Philips Healthcare). A two-dimensional T2^*^-weighted gradient-recalled echo sequence with long echo time, acquisition matrix size of 256 × 133–163 (emergency case: 224 × 109), slice thickness of 5 mm, and 1-mm spacing was used. CMBs were defined as being ≤ 10 mm in diameter on the basis of the previous study guidelines proposed by Greenberg *et al.* [16].

### Handgrip strength and definition of frailty

Handgrip strength was recorded with the participant standing and his/her arm extended in a natural position. The handgrip dynamometer (Smedley Dynamometer 0-1019-01; Matsumiya Ika Seiki Seisakujo, Tokyo, Japan) was adjusted for each participant, so that his/her second proximal phalanges were positioned around the handle. Handgrip strength was measured twice for both hands, and the maximum scores for both hands were analyzed.

We used Fried frailty phenotyping to assess the frailty of the participants [26]. The sum of the scores for the five components (unintentional weight loss, weakness, exhaustion, slowness, and low physical activity level) was treated as a continuous variable (positivity for each component was defined by a value of 1). Unintentional weight loss was defined as an unwanted loss of ≥ 3 kg over the preceding 6 months, determined at interview. Weakness was defined as a maximum handgrip strength less than the reference value for an Asian population [27]. Participants who were unable to perform the test were also considered to be weak. Exhaustion was determined using the Center for Epidemiologic Studies Depression Scale (CES-D) score. Slowness was determined using a 5-m walking test, and participants whose walking speed was ≤ 0.8 m/s were categorized as slow. Gait speed was measured using a standard test procedure and a stopwatch. Participants were instructed to walk 8 m at their normal speed, starting from a motionless standing position, and the time taken to traverse the space between meter three and meter eight was recorded. Low physical activity level was defined as an energy expenditure during physical activity of less than the reference value defined in a previous study of a Japanese population (≤ 6.20 kcal/kg/day for men and ≤ 7.13 kcal/kg/day for women) [28]. Every participant had their energy expenditure during physical activity assessed using a tri-axial accelerometer for 7 days, which was quantified as kcal/kg/day. The tri-axial accelerometer had to be worn for more than 480 min for the data to be included for that day, and data from participants with > 2 valid days were eligible for analysis.

### Clinical information

Body mass and height were measured while participants were wearing lightweight clothes and no shoes, and their body mass index (BMI) was then calculated. Systolic blood pressure (SBP) and diastolic blood pressure (DBP) were measured at rest using a blood pressure measuring device (HEM-907; Omron, Kyoto, Japan). The measurements were repeated when the SBP was ≥ 140 mmHg or the DBP was ≥ 90 mmHg, and the mean values were used in subsequent analyses.

Information regarding the age; sex; smoking status (never a smoker, former smoker, or current smoker); history of stroke, dementia, use of anti-hypertensive medication, diabetes mellitus, dyslipidemia, atrial fibrillation, CKD, or use of antiplatelet and/or anticoagulant medication; duration of education; household income (million yen per year); Mini-Mental State Examination score; and CES-D score were obtained by trained interviewers. The use of antiplatelet and/or anticoagulant medication was categorized as NOAC use (dabigatran, rivaroxaban, apixaban, or edoxaban) or warfarin use. Nutrient intake was assessed using a brief self-administered diet history questionnaire designed for Japanese adults [29].

Fasting blood samples were collected at the time of clinical examination into EDTA-2K tubes, siliconized tubes, and sodium fluoride tubes. Blood from the EDTA-2K tube was used to measure the white blood cell count and platelet count using an automated analyzer at SRL, Inc. (Tokyo, Japan). The serum concentrations of total protein, albumin, cystatin C, total cholesterol, high-density lipoprotein-cholesterol, LDL-cholesterol, triglycerides, glycated hemoglobin, and hs-CRP were measured using standard laboratory procedures.

### Statistical analysis

Demographic, clinical, laboratory, and other characteristics were summarized according to platelet count category (high or low). Differences in the mean values or proportions for each variable between platelet count categories were analyzed using the Wilcoxon rank sum test for continuous variables (age, height, body mass, BMI, handgrip strength, SBP, DBP, white blood cell count, platelet count, total protein, albumin, hs-CRP, cystatin C, glycated hemoglobin, total cholesterol, LDL-cholesterol, high-density lipoprotein-cholesterol, triglycerides, Mini-Mental State Examination score, CES-D score, daily energy expenditure during physical activity, daily energy intake, daily dietary protein intake, daily dietary vitamin D intake, daily dietary alcohol intake, and number of CMBs) or the McNemar chi-square test for categorical variables (sex, history of ischemic heart disease, use of anti-hypertensive medication, diabetes mellitus, dyslipidemia, atrial fibrillation, CKD, use of antiplatelet and/or anticoagulant medication, smoking status [never, former, and current smokers were defined as 1, 2, and 3, respectively], duration of education, household income, presence of CMBs, and frailty).

We performed a simple linear regression analysis to identify relationships between the presence of CMBs and handgrip strength or frailty. Next, to determine the relationships between the presence of CMBs and handgrip strength or frailty we used multivariable linear regression analysis for the full cohort and each platelet count category. In this analysis, *a priori* adjustments were made for age, sex, and BMI in Model 1, these plus classical cardiovascular risk factors (history of hypertension, history of diabetes mellitus, cystatin C, and smoking status) in Model 2, and these plus risk factors for sarcopenia (protein intake, vitamin D intake, and daily activity) in Model 3. These potential confounding factors were selected based on previous studies [1, 2, 14, 26]. We also performed a multivariable linear regression analysis to determine the relationships between the number of CMBs and handgrip strength or frailty. To assess the associations under consideration of the location of CMBs, we performed additional analyses using lobar CMBs (CMBs distributed in lobar and/or other locations) or non-lobar CMBs (CMBs distributed in deep or infratentorial and/or lobar locations).

As a sensitivity analysis, we performed multivariable linear regression analyses of the participants according to their platelet count category because the platelet count is an indicator of the capacity for endothelial repair, which might be associated with the maintenance of handgrip strength [5].

All analyses were two-tailed, and *p* < 0.05 was regarded as statistically significant. Statistical analyses were performed using STATA^®^ version 14.0 (StataCorp, College Station, TX, USA).

## Abbreviations

CMBs: cerebral microbleeds
MRI: magnetic resonance imaging
CKD: chronic kidney disease
NOACs: non-vitamin K antagonist oral anticoagulants
LDL: low-density lipoprotein
hs-CRP: high-sensitivity C-reactive protein
CES-D: Center for Epidemiologic Studies Depression Scale
BMI: body mass index
SBP: systolic blood pressure
DBP: diastolic blood pressure
